# Association between Consumption of Ultra-Processed Food and Body Composition of Adults in a Capital City of a Brazilian Region

**DOI:** 10.3390/nu15143157

**Published:** 2023-07-15

**Authors:** Larisse Monteles Nascimento, Layanne Cristina de Carvalho Lavôr, Bruna Grazielle Mendes Rodrigues, Felipe da Costa Campos, Poliana Cristina de Almeida Fonseca Viola, Massimo Lucarini, Alessandra Durazzo, Daniel Dias Rufino Arcanjo, Maria do Carmo de Carvalho e Martins, Karoline de Macêdo Gonçalves Frota

**Affiliations:** 1 Food and Nutrition Postgraduate Program, Department of Nutrition, Federal University of Piauí, Teresina 64049-550, PI, Brazil; 2 Health Sciences Center, Health and Community Postgraduate Program, Federal University of Piauí, Teresina 64049-550, PI, Brazil; 3 Department of Nutrition, Federal University of Piauí, Teresina 64049-550, PI, Brazil; 4 CREA—Research Centre for Food and Nutrition, Via Ardeatina, 546, 00178 Rome, Italy; 5 Department of Biophysics and Physiology, Federal University of Piauí, Teresina 64049-550, PI, Brazil

**Keywords:** ultra-processed foods, anthropometric, body composition, fat mass, lean mass

## Abstract

The present study investigates the relationship between the consumption of ultra-processed foods (UPF) and anthropometric indices of body composition in adults and seniors living in Teresina, the state capital of an area in northeastern Brazil. The article seeks to address two questions: Is UPF consumption linked to worsening body composition in different age groups? Do anthropometric indicators of body composition change with the increasing consumption of UPF? The study is a cross-sectional, household, population study, carried out with 490 adults and seniors. The food consumption was obtained with a 24 h food recall, and the foods were classified using NOVA. Anthropometric indicators evaluated were waist-to-height ratio, triceps skinfold thickness, arm circumference, corrected arm muscle area, subscapular skinfold thickness, and calf circumference. The association between energy contribution of UPF with anthropometric indicators was verified with a simple and multiple linear regression analysis. Individuals aged 20 to 35 years showed a significant association between UPF consumption and skinfold thickness (ß: 0.04; CI: 0.03/0.09), demonstrating an increase in this subcutaneous body fat marker with higher UPF consumption. Moreover, in participants aged 36 to 59 years, an inverse correlation between UPF intake and muscle mass markers, arm circumference (ß: −0.02; confidence interval: −0.03/−0.01), and corrected arm muscle area (ß: −0.07; confidence interval: −0.12/−0.02) were observed. Such results suggest there is decreased muscle mass with increasing UPF consumption. This is the first study that verified an association between UPF consumption and low-cost body composition indicators in different age groups.

## 1. Introduction

Due to the link between body fat and the likelihood of developing chronic diseases, determining body composition is crucial for the clinical practice and nutritional assessment of populations [1,2,3]. Anthropometric indicators are considered simple, accessible and non-invasive methods that can be used in clinical practice and in the context of epidemiological studies to classify individuals/populations regarding the risk of diseases related to excess and redistribution of fat [4,5].

In older adults, abdominal obesity, which is unrelated to overall obesity, increases the risk of developing chronic diseases [6]. In this sense, studies typically concentrate on nutrients, foods, or dietary patterns to find dietary factors linked to an increased risk of overweight and obesity [7,8,9]. Since advancements in food processing and technology have led to a rise in the availability, accessibility, and marketing of highly processed foods, the world’s food systems have seen significant changes in recent decades [10].

In this regard, researchers have created systems to group foods into categories based on their level of processing, from minimal to highly processed [11,12]. The NOVA categorization of foods [13,14,15] introduced by Monteiro et al. [13], which categorizes foods according to the purpose and level of processing to which they are exposed, is one of the most popular systems in the literature. Specifically, this classification divides foods into four categories: unprocessed or minimally processed foods; processed culinary ingredients; processed foods; and ultra-processed foods (UPF). The NOVA classification has been applied in different studies [16,17,18] as well as argumentations/criticism on this issue are ongoing [19,20,21]. UPF is heavy in sugar, salt, and additives, and it is lacking in dietary fiber and vitamins; typical examples of these energy-packed foods are snacks, soft drinks, candies, ready meals, refined starchy foods, and reconstituted meat products [22]. Therefore, the increase in consumption of “ultra-processed” foods may draw attention, for example, to the increase in consumption of toxic advanced glycation end-products (AGEs) obtained from the Maillard reaction during food processing, as many UPFs contain high levels of sugars and fats, favoring the formation of AGEs [23,24].

Evidence shows that just as AGEs may be associated transversally with loss of muscle mass in individuals over 45 years of age [25], UPF is also associated with changes in body composition measures, highlighting the increase in fat mass in the long term as well as the decrease in lean mass [26].

High UPF intake has been linked in several prospective cohorts and clinical trials to an increased risk of obesity [27], weight gain [28,29], and total and visceral fat accumulation [30]. Particularly, a recent meta-analysis of adults found that consuming UPF was linked to a markedly higher risk of 36% and 51% for overweight and obesity, respectively [31].

Techniques with high accuracy for estimating body fat, such as dual-energy X-ray absorptiometry (DXA), air displacement plethysmography, computed tomography, and magnetic resonance imaging, are expensive and difficult to implement at a population level. Accordingly, in clinical practice, other approaches such as anthropometric markers that distinguish body fat with low operational costs are required [32]. Anthropometric measurements are also part of the methods available to estimate body composition, where one can estimate the percentage of body fat and lean mass from information derived from skinfold measurements as well as using algorithms that account for gender, age, body mass, and height [33].

Therefore, it becomes essential to confirm the relationship between UPF intake and anthropometric markers, since there are still no studies showing this association.

## 2. Materials and Methods

### 2.1. Study Design and Population

This cross-sectional study was carried out in the state of Piauí’s cities of Teresina and Picos between the months of August 2018 and December 2019. This study is a component of the main project titled “Population-based Health Survey” (Inquérito de Saúde de Base Populacional, ISAD-PI).

Participants in this study ranged in age from 20 to 59 years old and 60 years or older from the city of Teresina, Piauí. Individuals living in private homes were eligible, except for those living in collective homes, pregnant women, and those with some disability or incapacity that made it impossible to carry out the survey.

### 2.2. Sample Size

The research sample was selected through a two-stage conglomerate sampling process: census sectors make up the main sampling units (PSU), while households make up the second stage, based on information from the IBGE census for the year 2010 [34].

The population size and the number of private houses in Teresina (767,557 people; 210,093 households) were taken into account while determining the sample size [34]. From these data, the average number of individuals for both genders per household was calculated in each of the following age groups: children under 2 years old; children aged 3 to 4 years; children aged 5 to 9 years; adolescents aged 10 to 14 years; adolescents aged 15 to 19 years; adults aged 20 to 59 and elderly over 6. A total of 578 households were then estimated to be enough. Using the formula *n* = *n*_0_/0.90 and assuming a response rate of 90%, the final sample size for this study was adjusted, yielding *n* = 642 households, approximately. Further details about the sample size and sampling plan were previously published by Rodrigues et al. [35]. Using the same sampling strategy, 50% of the households were chosen to form a subsample for the purpose of gathering information on food consumption using 24 h recalls.

**Figure 1:**
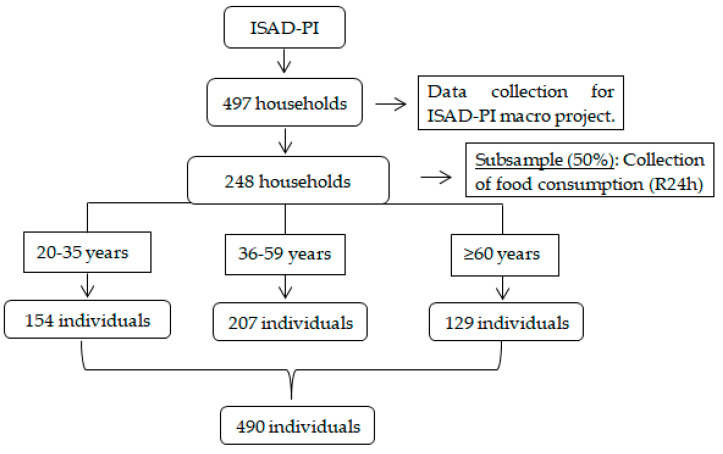
Study sample flowchart. Abbreviation: ISAD-PI, Population-Based Health Survey; R24h: 24 h food recall.

Upon completion of the study, 497 Teresina households were included in the final sample, and the subsample consisted of 248 households. In the end, data were obtained from 490 individuals, with 154 participants aged 20 to 35 years, 207 aged 36 to 59 years, and 129 individuals aged 60 years or over (Figure 1). This study was approved by the Committee for Ethics in Research from the Federal University of Piauí, under decision n° 2.552.426.

### 2.3. Data Collection and Anthropometric Measurements

The National Health Survey, conducted in 2013 by the Brazilian Institute of Geography and Statistics [36], and other Brazilian studies, including “ISA 2008: Health Surveys in the city of São Paulo” [37], were both used as models for the structured questionnaires that trained researchers used to collect demographic, socioeconomic, anthropometric, lifestyle, and food consumption data. The data were collected using mobile devices and the Epicollect 5^®^ application (Imperial College London; https://five.epicollect.net/project/isad, accessed on 3 April 2023).

Anthropometric measurements were estimated in accordance with recommendations by Cameron [38] as well as Jelliffe and Jelliffe [39]. The participants were weighed using a portable electronic scale (SECA^®^, model 803, Hamburg, Germany) with a precision of 100 g, and their heights were measured using a stadiometer (SECA^®^, model messband 206, Hamburg, Germany) with a precision of 0.1 cm. Adults’ Body Mass Index (BMI) was categorized in accordance with the World Health Organization’s guidelines [40]; for elderly individuals, the values established by Lipschitz et al. [41] were used.

According to WHO recommendations [42], waist circumference (WC) was measured using an inelastic measuring tape with a precision of 0.1 cm at the midpoint between the last rib and the iliac crest. The waist-to-height ratio (WHtR) was calculated by dividing WC in centimeters by height, also in centimeters [43].

According to Frisancho [44], the arm circumference (AC), or the distance between the acromion and the olecranon on the right arm, was measured in order to calculate the arm muscle circumference (AMC) and the arm muscle area (AMA).

AMC was calculated from the formula: AC-(π*TSF) in centimeters. In turn, AMA was calculated using the formula: AMA = (AMC2/4*π). The AMA correction (CAMA) was 10 for men and 6.5 for women.

The triceps (TSF) and subscapular skinfolds were measured using an adipometer (Lange^®^, Cambridge Scientific Industries, Inc., Cambridge, MA, USA) calibrated with an accuracy of 0.1 mm. The TSF measurement was taken on the posterior surface of the right arm, parallel to the longitudinal axis, at the midpoint between the acromion and the olecranon. The measurement of the subscapular skinfold (SSF) was estimated only in adults, on the right side of the individual, and two centimeters below the inferior angle of the scapula [45].

Calf circumference (CC) was measured only in elderly individuals with an inelastic measure tape with a precision of 1 mm positioned in the largest volume of the left calf, with the individual in a chair with the leg flexed at 90° [46]. Values below 31 cm were considered indicative of loss of muscle mass [42].

### 2.4. Dietary Assessment

The food consumption of the studied population was obtained by applying the 24 h food recall (R24h) based on the multiple pass method, which proposes five steps for collecting dietary data: 1st stage—quick listing of foods and schedules; 2nd stage—filling out the commonly forgotten foods; 3rd stage—setting the schedule and naming the meals; 4th stage—detailing and reviewing the meals, including the amount eaten, form of preparation, origin, commercial brand, portion size, including the addition of salt, sugar, butter, or margarine to foods and preparations; 5th stage—final review of the R24h. Should be reviewed the foods whose reporting was difficult, if R24h is complete and ask about the consumption of alcoholic beverages, snacks and at social events [47,48].

In order to correct intrapersonal variability, a second R24h was administered to 40% of the population after a two-month break, using the identical techniques from the first interview. Based on a study by Verly-Júnior et al. [49], it was determined that regardless of sample size, the application of a second R24h in 40% of the sample resulted in no decrease in precision for predicting food intake.

The Table for Measuring Household Food Consumption [50] was used to convert the household measures obtained from the interviews into grams (g) or milliliters (mL). The Brazilian Table of Food Composition [51], Table of Nutritional Composition of Foods Consumed in Brazil [52], and Table of Food Composition: Support for Nutritional Decision [53] were used to compute the energy intake.

Using the NOVA classification, the reported food items were divided into 4 groups based on the degree of processing they underwent [14]. Foods in groups 1, 2, and 4 were minimally or not at all processed, treated for use in cooking, and extremely processed. The first and fourth groups, however, were the only ones examined in this study.

Once removed from nature, unprocessed foods include edible parts of plants or animals, as well as mushrooms, algae, and water. These edible parts include those from plants (such as seeds, fruits, leaves, and roots) or animals (such as muscles, eggs, and milk). Fresh foods that have undergone minimal processing include those that have had undesirable or inedible parts removed, were dried, dehydrated, ground or milled, fractioned, roasted, pasteurized, cooled or frozen, vacuum-packed, gone through non-alcoholic fermentation, and/or gone through other processes without having salt, sugar, oils, or fats added. For example, wheat grains are converted into products like flour, couscous, and pasta [14].

UPF is in group 4, and its industrial formulations often contain five chemicals or more. These ingredients frequently include preservatives, stabilizers as well as other ingredients or additions used in the production of processed meals such as salt, sugar, oils, and fats. UPF is frequently found in products including soft drinks, rehydration supplements, “packaged snacks”, ice cream, and chocolate, among others [14]. The Total Energy Value (TEV) of the study participants’ diets was used to calculate the proportion of each food group’s consumption. All food consumption analyses were carried out using Stata software (version 13.0).

### 2.5. Statistical Analysis

The Stata software, version 13.0, was used to analyze the data. In order to compensate for the complex sampling of this research, all analyses were carried out in survey mode. Standard deviation and average were used to express continuous variables, whereas absolute and relative values were used to convey categorical variables. Using the Shapiro–Wilk test, the distributions of continuous variables were examined. In turn, in order to make comparisons among averages related to the Student’s *t*-test and ANOVA, a variance analysis was used, followed by Tukey’s post-hoc test. The association between the energy contribution of UPF consumption and anthropometric indicators was verified using simple and multiple linear regression analyses that were corrected for potential confounding variables. The independent variables included in the modified model were selected by creating the Directed Acyclic Graph (DAG) in the software Daggity, version 3.0, in order to control for confounding questions (Figure S1). In accordance with the backdoor criterion [54], it was identified that there was a need for minimal adjustment for gender, education, family income, marital status, alcohol consumption, smoking, practice of physical activity, BMI, and WC. A 5% level of significance and 95% confidence intervals were used.

## 3. Results

A total of 490 people took part in the study, with the majority (76.3%) earning less than or equal to two minimum wages, being between the ages of 36 and 59 as well as 42.2% of them being female. In addition, when it came to lifestyle choices, 80.4% of people were active, 78% did not smoke, and 60% did not consume alcoholic beverages. There was a greater participation of fresh or minimally processed foods in relation to the average percentage of contribution in the Total Energy Value (TEV) for the NOVA classification food groups (67.9% of the TEV 18.9); however, the UPF group obtained an expressive contribution in terms of TEV (19.7% of the TEV 17.9) (Table 1).

**Figure 2:**
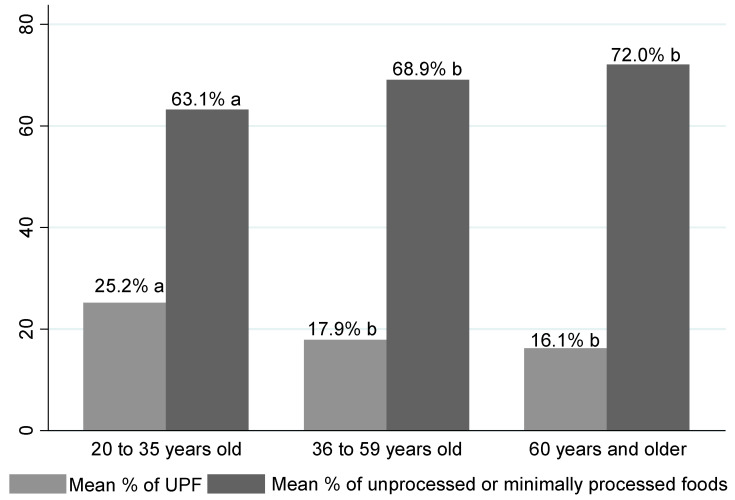
Mean percentage contribution to TEV from consumption of ultra-processed foods and fresh foods by age group. Equal letters do not differ statistically. Different letters indicate significant differences between means.

The average percentage of the contribution in terms of the TEV of UPF consumption and fresh or minimally processed foods according to age group is displayed in Figure 2; it is noted (Figure 2) that UPF consumption was significantly higher for the group of individuals aged 20 to 35 years (25.2%) when compared to the participants aged 36 to 59 years (17.9%) and over 60 years (16.1%). With regard to the consumption of fresh or minimally processed foods, the younger group (20 to 35 years old) again maintained a less healthy profile with an intake of 63.1%, compared to 68.9% and 72.0%, consumed by adult individuals aged 36 to 59 years and elderly individuals, respectively (Figure 2).

Table 2 shows the distribution of anthropometric indicators according to age group. The waist-to-height ratio increased significantly with age; however, TSF decreased when comparing the group aged 36 to 59 years (25.1 ± 10.4) and the elderly population (21.1 ± 8.7) (Table 2). Furthermore, for the AC measurement, a significant increase in individuals aged 20 to 35 years (31.2 ± 4.8) with respect to the group aged between 36 to 59 years (32.2 ± 4.7) was observed, whereas a reduction in this measurement with increasing age (30.3 ± 4.6) in the elderly population was observed. As reported in Table 2, subscapular skinfold thickness (SST) increased with advancing age when comparing the groups aged 20 to 35 years (21.4 ± 9.0) and individuals aged 36 to 59 years (23.7 ± 9.5).

The analyses of the association between UPF consumption and anthropometric indicators according to age group are reported in Table 3. In the group of individuals aged 20 to 35 years, there was a significant association between UPF consumption and TSF (ß: 0.04; CI: 0.03/0.09; *p* = 0.04), thus demonstrating an increase in this subcutaneous fat marker with a higher UPF consumption. Furthermore, for participants aged 36 to 59 years, there was an inverse and significant association between UPF consumption and the muscle mass markers AC (ß: −0.02; CI: −0.03/−0.01; *p* = 0.03) and CAMA (ß: −0.07; CI: −0.12/−0.02; *p* = 0.01) were, respectively, observed. Such results suggest a decrease in muscle mass indirectly measured with the AC and CAMA parameters with the increase in UPF consumption.

## 4. Discussion

This study explored the relationship between UPF intake and anthropometric measurements of body composition. These methodologies are used in population studies and in clinical practice for estimating fat and lean body mass at low cost. The greater contribution of UPF to the diet was associated with increased markers of subcutaneous fat and decreased markers of lean body mass, for adults aged 20 to 35 years and for those aged 36 to 59 years, respectively. These findings demonstrate that a higher UPF consumption entails a negative impact on the body composition of adults in different age groups, thus contributing to an increase in subcutaneous fat as a risk factor for the emergence of CNCD.

The contribution of UPF consumption in this research was greater for younger adults, and it was also observed that the consumption of this food group decreased with increasing age. Such results corroborate those by Costa et al. [55], who showed that, in the population of 27 Brazilian capitals, the frequency of UPF consumption decreased linearly with age. This evidence may be due to the greater concern of older and long-living adults with health, in addition to the possibility that most of them have more consistent eating patterns and conventional culinary techniques. Furthermore, along with having less stable dietary habits and being more willing to test out these items, these population groups might also be less susceptible to marketing strategies that appeal to the younger generation [56].

The increase in body fat among the participants of this research was observed through waist-to-height ratio (WHtR) and subscapular skinfold thickness (SST) measurements with advancing age. On the other hand, among the elderly individuals, a loss of fat mass was observed, based on the TSF levels’ observations, as well as a loss of lean mass through CAMA levels. In old age, the body composition changes as fat mass increases and redistributes. Fat-free mass consisting mainly of skeletal muscle decreases by 40% between the ages of 20 and 70 years, and after age 70, both fat mass and lean mass decrease together. Accordingly, with the redistribution of fat mass, an increase in visceral and muscular fat deposits takes place [57,58].

A growing body of research indicates that consuming UPF increases the risk of obesity [59]. UPF consumption is strongly correlated with obesogenic dietary nutritional profiles, according to analyses of nationally representative dietary surveys carried out in numerous nations, including the United Kingdom [60]. Recent population-based cross-sectional studies in Brazil [61], the United States [62], and Canada [63] have shown a positive correlation between eating ultra-processed foods and obesity. Additionally, a two-week randomized controlled crossover study of 20 adults in weight stability found that eating more ultra-processed food resulted in higher energy intake, a significant increase in body weight, and a rise in fat mass [28].

This is the first research with the aim of investigating the influence of UPF consumption on body composition, assessed using anthropometric indicators. The association presented in this study highlighted that higher UPF consumption may reflect an increase in fat mass through the increase in terms of TSF in adults aged 20 to 35 years. Similar results were verified by Liu et al. [64], who—despite using a more sensitive method, such as dual-energy X-ray absorptiometry (DXA), in order to measure the body composition of adults aged between 20 and 59 years—also observed that higher UPF consumption was associated with increased body fat in a population sample assessed in the United States.

Furthermore, the reduction in lean body mass was observed in adults aged 36 to 59 years in this study when highlighting an inverse association between UPF consumption and AC and CAMA measurements. The study by Viola et al. [65] with Brazilian adolescents also demonstrated the association between the decrease in muscle mass measured using a DXA and the increase in the participation of UPF consumption in the diet. The unbalanced nutritional composition of foods and beverages favors increased energy intake, a reduction in protein intake, and an increase in nutrients/ingredients of low nutritional quality, such as refined sugars. The AGEs present in UPF are one mechanism that, according to scientific evidence, promotes changes in body composition [13,24,25,66]. The presence of food additives and contaminants created during processing alters the profile and composition of the intestinal microbiota [67], and changes in the food matrix induced by food processing seem to influence the kinetics of nutrient absorption and changes in the gut–brain signaling of satiety, among other unclear mechanisms that are interrelated, and promote inflammation, oxidative stress, and ensuing weight gain [26,28,62].

Accordingly, considering the results of this study that show an association between UPF consumption and increased body fat and decreased lean mass, the importance of further research investigating the association between this food group and body composition is reiterated and assessed using accessible anthropometric indicators that can be easily used in clinical practice. In addition, it is important to raise awareness about reducing UPF consumption, which is the second most consumed food group by the Brazilian population, as they are convenient formulations, often sold in large portions, aggressively marketed, and extremely tasty [68].

Some limitations of this study are important to be highlighted, such as its cross-sectional design, which limits the relationships between cause and effect. Moreover, when using the 24 h dietary recall, some inconsistencies can be identified, partially due to stated consumption errors (over or underreporting), in part. However, two recalls were employed: the first applied to the complete population, and the second applied to 40% of this population, with the purpose of correcting intrapersonal variability in order to minimize these inaccuracies [49]. Among the strengths of this research, originality stands out since it is the first study to show an association between the consumption of ultra-processed foods and anthropometric indicators in adult and elderly people.

## 5. Conclusions

This study provides significant research demonstrating links between UPF consumption, obesity, and measures of lean body mass, thus indicating the negative influence of these foods on body composition in the population. In addition, these findings indicate the need to take policy initiatives, especially with regard to the labeling of UPF with an emphasis on the obligation supported by legislation, in the sense of informing the consumer about the real harm to health that the replacement of natural or minimally processed foods with UPF can cause. Such information could be presented through a QR code present on UPF packaging, emphasizing that reduction in terms of UPF consumption should be considered, as the high consumption of this food group can be harmful to health.

## Figures and Tables

**Table 1:** Characterization of the study population.

Age (Years)	*n* (%)
20–35	154 (31.4)
36–59	207 (42.2)
≥60	129 (26.3)
Gender	
Masculine	162 (33.1)
Feminine	328 (66.9)
Marital status	
Single	286 (58.5)
Married	203 (41.5)
Family income (Brazilian minimum wage)	
≤2	374 (76.3)
>2	116 (23.7)
Level of education	
Not literate	29 (5.9)
Primary education	149 (30.4)
High school	201 (41)
University education	111 (22.7)
Alcohol consumption	
No	294 (60)
Yes	196 (40)
Smoking	
No	382 (78)
Yes	108 (22)
Physical activity	
Insufficiently active	96 (19.6)
Active	394 (80.4)
Mean contribution percentage in the TEV of the food groups NOVA	
Food groups NOVA	Mean ± SD
Unprocessed or minimally processed	67.9 ± 18.9
Ultra-processed food	19.7 ± 17.9

TEV: Total Energy Value.

**Table 2:** Distribution of anthropometric indicators according to age group.

	Mean ± SD	20–35 Years	36–59 Years	≥60 Years
WHtR	0.56 ± 0.81	0.52 ± 0.07 ^a^	0.57 ± 0.07 ^b^	0.61 ± 0.07 ^c^
TSF	23.7 ± 10.1	23.9 ± 10.6 ^ab^	25.1 ± 10.4 ^a^	21.1 ± 8.7 ^b^
AC	31.4 ± 4.6	31.2 ± 4.8 ^a^	32.2 ± 4.7 ^b^	30.3 ± 4.6 ^a^
AMC	23.9 ± 3.6	23.7 ± 3.9 ^a^	24.3 ± 3.7 ^a^	23.7 ± 3.0 ^a^
CAMA	39.1 ± 13.7	38.3 ± 14.6 ^a^	40.6 ± 14.5 ^a^	37.8 ± 10.8 ^a^
SSF	22.7 ± 9.3	21.4 ± 9.0 ^a^	23. 7 ± 9.5 ^b^	-
CC	34.1 ± 3.6	-	-	-

SD: standard deviation; WHtR: waist-to-height ratio; TSF: triceps skinfold; AC: arm circumference; AMC: arm muscle circumference. AMC: AC(cm) − (3.14 × TSF); CAMA: arm muscular area corrected; SSF: subscapular skinfold; CC: calf circumference. Equal letters do not differ statistically. Different letters indicate a significant difference between means.

**Table 3:** Association between consumption of ultra-processed foods and anthropometric indicators according to age group (*n* = 490).

	ß (CI95%)	*p*	ß (CI95%)	*p* *
20–35 years (*n* = 153)				
WHtR	−0.03 (−0.08/0.02)	0.27	−0.01 (−0.02/0.05)	0.19
TSF	0.03 (−0.07/0.12)	0.54	0.04 (0.03/0.09)	0.04
AC	−0.00 (−0.05/0.04)	0.82	−0.01 (−0.01/0.02)	0.38
AMC	−0.01 (−0.04/0.01)	0.21	−0.01 (−0.02/0.01)	0.49
CAMA	−0.05 (−0.13/0.03)	0.22	−0.02 (−0.08/0.04)	0.54
SSF	−0.02 (−0.09/0.06)	0.65	−0.003 (−0.05/0.04)	0.89
36–59 years (*n* = 207)				
WHtR	0.02 (−0.06/0.06)	0.92	−0.03 (−0.02/0.02)	0.76
TSF	0.04 (−0.05/0.13)	0.36	−0.00 (−0.05/0.04)	0.92
AC	−0.01 (−0.05/0.03)	0.63	−0.02 (−0.03/−0.01)	0.03
AMC	−0.02 (−0.04/−0.00)	0.04	−0.02 (−0.03/−0.00)	0.01
CAMA	−0.08 (−0.16/−0.006)	0.04	−0.07 (−0.12/−0.02)	0.01
SSF	0.03 (−0.03/0.10)	0.32	0.01 (−0.04/0.06)	0.65
≥60 years (*n* = 128)				
WHtR	0.00 (−0.01/0.01)	0.89	−0.03 (−0.04/0.02)	0.80
TSF	0.03 (−0.06/0.13)	0.49	−0.02 (−0.07/0.04)	0.59
AC	0.01 (−0.03/0.06)	0.52	0.01 (−0.004/0.03)	0.13
AMC	0.00 (−0.03/0.03)	0.77	0.02 (−0.002/0.04)	0.08
CAMA	0.03 (−0.08/0.13)	0.61	0.06 (−0.01/0.13)	0.10
CC	−0.0005 (−0.04/0.04)	0.98	0.006 (−0.02/0.03)	0.61

CI: confidence interval; WHtR: waist-to-height ratio; TSF: triceps skinfold; AC: arm circumference; AMC: arm muscle circumference. AMC: AC(cm) − (3.14 × TSF); CAMA: arm muscular area corrected; SSF: subscapular skinfold; CC: calf circumference. * Adjusted for gender, schooling, family income, marital status, alcohol consumption, smoking, practice of physical activity, BMI, and WC.

## Data Availability

The authors declare that the data supporting the findings of this study are available within the article. All other data are available on reasonable request from the corresponding authors.

## Supplementary Materials

The following supporting information can be downloaded at: https://www.mdpi.com/article/10.3390/nu15143157/s1. Figure S1: Directed Acyclic Graph (DAG).
