# Supplementary figures and images for: Association between Consumption of Ultra-Processed Food and Body Composition of Adults in a Capital City of a Brazilian Region
Source: Nutrients. 2023 Jul 15;15(14):3157. doi: 10.3390/nu15143157 (PMC10383416; doi:10.3390/nu15143157)

## Directed Acyclic Graph (DAG)

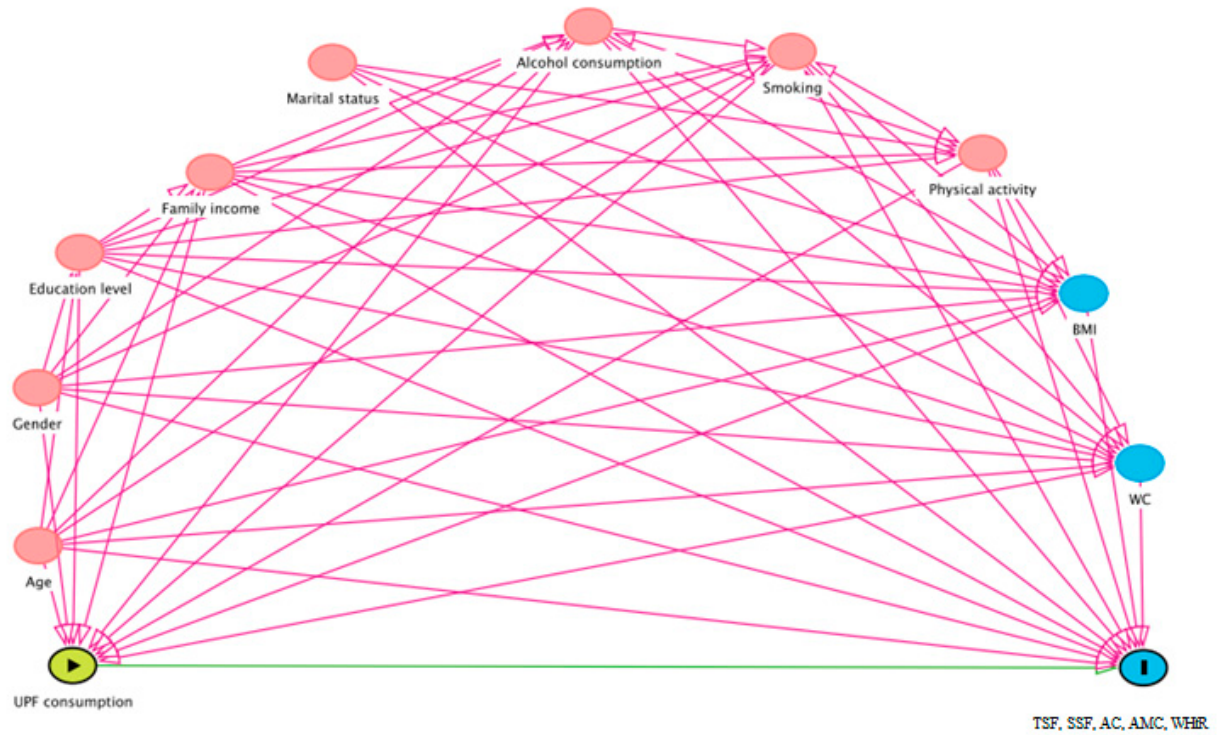

Supplement: Supplementary file 1 [file nutrients-15-03157-s001.zip › nutrients-2489003-supplementary.pdf]
